# Classic serotonergic psychedelics for mood and depressive symptoms: a meta-analysis of mood disorder patients and healthy participants

**DOI:** 10.1007/s00213-020-05719-1

**Published:** 2021-01-11

**Authors:** Nicole L. Galvão-Coelho, Wolfgang Marx, Maria Gonzalez, Justin Sinclair, Michael de Manincor, Daniel Perkins, Jerome Sarris

**Affiliations:** 1 Laboratory of Hormone Measurement, Department of Physiology and Behavior, Federal University of Rio Grande do Norte, Natal, RN Brazil; 2 Postgraduate Program in Psychobiology and Department of Physiology and Behavior, Federal University of Rio Grande do Norte, Natal, RN Brazil; 3 National Institute of Science and Technology in Translational Medicine, São Paulo, Brazil; 4 NICM Health Research Institute, Western Sydney University, Westmead, Australia; 5 Departamento de Fisiologia e Comportamento, Universidade Federal do Rio Grande do Norte, Caixa Postal, 1511, CEP: 59078-970 Natal, RN Brasil; 6 IMPACT Research Institute, School of Medicine, Deakin University, Geelong, Australia; 7 School of Social and Political Science, University of Melbourne, Melbourne, Australia; 8 Professorial Unit, The Melbourne Clinic, Department of Psychiatry, University of Melbourne, Melbourne, Australia

**Keywords:** Depression, Psilocybin, Ayahuasca, LSD, Mescaline, Placebo

## Abstract

**Rationale:**

Major depressive disorder is one of the leading global causes of disability, for which the classic serotonergic psychedelics have recently reemerged as a potential therapeutic treatment option.

**Objective:**

We present the first meta-analytic review evaluating the clinical effects of classic serotonergic psychedelics vs placebo for mood state and symptoms of depression in both healthy and clinical populations (separately).

**Results:**

Our search revealed 12 eligible studies (*n* = 257; 124 healthy participants, and 133 patients with mood disorders), with data from randomized controlled trials involving psilocybin (*n* = 8), lysergic acid diethylamide ([LSD]; *n* = 3), and ayahuasca (*n* = 1). The meta-analyses of acute mood outcomes (3 h to 1 day after treatment) for healthy volunteers and patients revealed improvements with moderate significant effect sizes in favor of psychedelics, as well as for the longer-term (16 to 60 days after treatments) mood state of patients. For patients with mood disorder, significant effect sizes were detected on the acute, medium (2–7 days after treatment), and longer-term outcomes favoring psychedelics on the reduction of depressive symptoms.

**Conclusion:**

Despite the concerns over unblinding and expectancy, the strength of the effect sizes, fast onset, and enduring therapeutic effects of these psychotherapeutic agents encourage further double-blind, placebo-controlled clinical trials assessing them for management of negative mood and depressive symptoms.

**Supplementary Information:**

The online version contains supplementary material available at 10.1007/s00213-020-05719-1.

## Introduction

### Overview

There is currently a resurgence of research investigating the use of psychedelic substances in the treatment of mood disorders, mainly the “classic serotonergic psychedelics” (Chi and Gold 2020; Dos Santos et al. 2018; Reiff et al. 2020; Schenberg 2018). This class of psychedelics includes psilocybin, dimethyltryptamine ([DMT], often consumed via the traditional plant preparation ayahuasca), lysergic acid diethylamide (LSD), and mescaline. These substances may induce psychedelics effects as potential agonists of the serotonin 2A receptors. Although it is recognized, they may also interact to a lesser extent with other neurotransmitter pathways, such as partial agonism of the serotonin 2C receptors, through which they may also induce antidepressant activity (Araujo et al. 2015; Baumeister et al. 2014; Halberstadt and Geyer 2011).

One factor contributing to this resurgence is the limited success of existing pharmacotherapies for patients with depressive disorders (Thase et al. 2001). Many antidepressants may have a long latency to therapeutic response, requiring two to 6 weeks to produce effects, and can potentially induce undesirable side-effects, resulting in increased patient distress or discontinuation of treatment (Blier and de Montigny 1994; Carvalho et al. 2016; Posternak and Zimmerman 2005). A substantial portion of depressed patients do not benefit substantially from antidepressant treatment, (Cipriani et al. 2018; Hengartner and Plöderl 2018; Kirsch 2014; Munkholm et al. 2019; Thase et al. 2001). As a result, up to half of patients with depression may develop treatment-resistant disorders, defined as a failure to achieve remission with two or more adequate antidepressant trials (Akil et al. 2018; Conway et al. 2017).

Given the scale and impact of this problem, innovative treatment approaches for major depression are urgently needed, and it is in this context that there has been renewed clinical and research interest in the classic serotonergic psychedelics (Dos Santos et al. 2018; Schenberg 2018). In general, serotonergic psychedelics do not lead to withdrawal or compulsive drug-seeking behaviors, as are observed with substances such as opioids and cocaine (Bogenschutz and Johnson 2016; McKenna 2004). They are generally considered safe and do not induce physiological toxicity or lasting adverse effects, although transitory signs of cognitive and emotional alterations, and mild sympathetic activity, are common (Bogenschutz and Ross 2018). Studies examining data from the National Survey on Drug Use and Health (2001–2004) in the USA have reported no significant associations between lifetime psychedelic use and adverse mental health outcomes, including psychosis, with some evidence of reduced risk of these outcomes (Johansen and Krebs 2015; Krebs and Johansen 2013). However, restrictions on the use of psychedelics are indicated for individuals with severe cardiac disease and either a personal or family history of psychosis.

Therefore, the classic psychedelics are again being utilized in pre-clinical, observational, open-label, and randomized controlled trials examining effects on mood in psychiatry patients and healthy volunteers, with highly encouraging initial results being reported (Carhart-Harris et al. 2016b; Dolder et al. 2016; Dos Santos et al. 2011; Hasler et al. 2004; Kometer et al. 2012; Kraehenmann et al. 2015; Osorio Fde et al. 2015; Palhano-Fontes et al. 2019; Ross et al. 2016; Schmid et al. 2015; Schmid and Liechti 2018; Wittmann et al. 2007). Mescaline, usually derived from the peyote cactus (Bogenschutz and Ross 2018; Heffter 1898), and LSD were investigated in older psychiatric studies, conducted to explore effects on psychosis and alcoholism (Berlin et al. 1955; Blum et al. 1977; Fuentes et al. 2019; Gouzoulis-Mayfrank et al. 1998; Hofmann 1979; Krebs and Johansen 2012; Pahnke et al. 1970; Rucker et al. 2018). More recently, LSD studies have turned to the investigation of its effect on mood (Carhart-Harris et al. 2016b; Dolder et al. 2016; Gasser et al. 2014; Gasser et al. 2015; Schmid et al. 2015; Schmid and Liechti 2018).

The current second wave of psychedelic research has primarily involved psilocybin, the main psychedelic compound of *Psilocybe* spp. fungi (Rucker et al. 2018). Open-label trials have been conducted in obsessive-compulsive disorder (Ballenger 2008; Moreno et al. 2006), addiction (Bogenschutz et al. 2015; Johnson et al. 2014), and treatment-resistant depression (Carhart-Harris et al. 2016a; Carhart-Harris et al. 2017), whereas double-blind trials have been conducted in patients with life-threatening cancer diagnoses (commonly exploring effects on mood and existential anxiety) (Griffiths et al. 2016; Griffiths et al. 2006; Grob et al. 2011; Hasler et al. 2004; Kometer et al. 2012; Kraehenmann et al. 2015; Ross et al. 2016; Wittmann et al. 2007). On the strength of this evidence, the United States Food and Drug Administration granted “breakthrough therapy” status to psilocybin in 2019, concluding that initial data indicate that it may provide a substantial improvement over existing treatments for treatment-resistant depression (Pathways 2018).

Ayahuasca is an Amazonian brew made with *Psychotria viridis*, a rubacea containing N, N-DMT, and *Banisteriopsis caapi*, a vine which contains β-carbolines that are reversible inhibitors of monoamine oxidase and an inhibitor of serotonin reuptake (Palhano-Fontes et al. 2014). This psychedelic also has been subject to increasing research, which has included effects on mood and addiction in healthy volunteers (McKenna 2004; Riba et al. 2001; Santos et al. 2007; Uthaug et al. 2018), as well as clinical trials investigating its psychological and neurobiological antidepressant effects in open-label and double-blind designs (de Almeida et al. 2019; Dos Santos et al. 2011; Galvão et al. 2018; Osorio Fde et al. 2015; Palhano-Fontes et al. 2019; Zeifman et al. 2019).

### Aims and objective

As a consequence of the expanding number of clinical studies investigating psychedelic treatments for psychiatric disorders, the number of reviews on this topic has also increased in recent years (Bogenschutz and Ross 2018; Chi and Gold 2020; Dos Santos et al. 2018; Johnson et al. 2019; Muttoni et al. 2019; Ross 2018; Rucker et al. 2018). A recent meta-analysis review showed that psychedelic-assisted therapy, which included both the classic serotonergic psychedelics and MDMA (3,4-methylenedioxymethamphetamine), significantly outperformed placebo, with large effect sizes across a range of mental disorders such as unipolar depression, anxiety, and post-traumatic stress disorder (Luoma et al. 2020). However, to date, no meta-analysis of double-blind randomized controlled trials (RCTs) comparing the clinical efficacy of classic serotonergic psychedelics with placebo, for mood and depressive symptoms, has been published. Our aim is to present the first meta-analysis in this area, by evaluating the clinical effect of classic serotonergic psychedelics on negative mood state and depressive symptoms, in double-blind RCTs, separately for both healthy volunteers and patients diagnosed with a mood disorder.

## Methods

The search strategy and data synthesis were conducted in line with the Preferred Reporting Items for Systematic Reviews and Meta-Analyses (PRISMA) statement (Moher et al. 2009) and followed a registered protocol (PROSPERO registration number: CRD42020158356).

### Systematic search

The systematic search was conducted using the Cochrane Central Register of Controlled Trials, Cochrane Database of Systematic Reviews, Health Technology Assessment Database, Allied and Complementary Medicine, PsycINFO, and Ovid MEDLINE(R), from journal inception to May, 2020. A search via Web of Science was conducted using the same keywords to identify any additional relevant articles. Reference lists of included articles were also searched. During the initial screening, 4 raters (NLGC, MG, JS, and MM) independently assessed articles retrieved for eligibility based on the title, abstract, and in method, after which full text articles were retrieved and screened.

### Eligibility criteria

Eligibility criteria were organized in accordance with the PICO (participants, interventions, comparisons, and outcomes) reporting structure, as described below. The search terms used in systematic review search are summarized in Table 1.

**Table 1:** Search terms used in systematic review search

Participants	
Human clinical trials involving patients with depression or healthy volunteers	
Interventions	
Psychedelic* or Lysergic acid or LSD or Dimethyltryptamin* or DMT or Ayahuasca or Hoasca or Psilocybin or Mescaline or Peyote.	
Comparator	
Random* or Trial or Intervention	
Outcomes	
Depression or Depressive Mood or Mental Illness or Mental Health or Mental Disorder or Affective Disorder	

### Participants

After a systematic search of the relevant data, we included studies with both healthy individuals and patients with mood disorders who were diagnosed using the Diagnostic and Statistical Manual of Mental Disorders IV (DSM-IV) (Table 1). The data for patients with a mood disorder and healthy participants were analyzed separately to avoid conflating heterogeneous populations.

### Interventions

All classic serotonergic psychedelic interventions were included: mescaline, LSD, DMT/Ayahuasca, and psilocybin. However, after a systematic search, we included studies that investigated psychedelic interventions using the “psychedelic model,” where interventions were provided to participants in moderate to high doses in single or multiple sessions (Reiff et al. 2020; Ross 2012). Studies using micro-doses and low doses of psychedelics (e.g., for psilocybin below 100 μg/kg) (Hasler et al. 2004) were excluded.

### Comparation

All studies included were randomized, placebo-controlled, and double-blind trials, which had either a cross-over or parallel design, with outcomes between 3 h and 60 days after dosing session (see below the “Outcomes” section for more details). Control groups must have been an inactive comparator such as a placebo, low-dose psychedelic, or a non-psychoactive pharmacological agent (e.g., niacin).

### Outcomes

We included trials that used clinically validated scales for depression or mood state outcomes. For depression symptoms, those scales were as follows: Hamilton Depression Rating Scale (HAM-D), Montgomery–Åsberg Depression Rating Scale (MADRS), Beck Depression Inventory (BDI), and Hospital Anxiety and Depression Scale (HADS; specifically, the depressive sub-score). To measure mood state, the following scales were included: Adjective Mood Rating Scale (AMRS; specifically anxiety-depressiveness sub-score), Positive and Negative Affect Schedule (PANAS; specifically negative score), Profile of Mood States (POMS), and Persisting Effects Questionnaire (PEQ; specifically negative mood).

To provide temporal clarity of the psychedelic’s effects (Carhart-Harris et al. 2016b), the outcomes of mood state and depressive symptoms were categorized and analyzed with respect to time-point collected after the respective dosing session:

- Acute effects: outcomes from 3 h to 1 day after dosing session.
- Medium-term effects: outcomes from 2 to 15 days after dosing session.
- Long-term effects: outcomes from 16 to 60 days after dosing session.

These time-points were selected because antidepressant drugs commonly require approximately 2 weeks to initiate a therapeutic response (Cipriani et al. 2018; Hengartner and Plöderl 2018), while the psychotropic effects of substances with psychedelic actions, for instance ketamine and classical serotonergic psychedelics, commence within hours and have been reported to last approximately 2 weeks (Corriger and Pickering 2019; Sanacora et al. 2017).

We excluded data from studies with outcomes measured within less than 3 h of receiving the psychedelic to avoid assessment confounding from changes in cognition and perception induced acutely by the substance. Furthermore, data was also excluded from trials with data collected 60 days or longer after the intervention due to the poor reliability of retrospective data. Outcomes from the open-label phase of double-blind studies were also not included. There was no restriction based on sample size, the duration or severity of symptoms, comorbid disorders, or participant demographics.

### Quality assessment of included clinical trials

The quality of eligible clinical trials was assessed using the Jadad scale (Jadad et al. 1996) completed by author NLGC and cross-checked by WM and JS.

### Data extraction and analysis

Effect size data of each experimental group was extracted and converted to standardized mean differences (SMD) with 95% confidence intervals (CIs). Data were initially extracted by one author (NLGC), and then cross-checked independently by an additional author (WM). In line with conventional interpretations, SMD were classified as negligible (< 0.2), small (0.2–0.4), moderate (0.4–0.8), or large (> 0.8) (Higgins and Green 2011).

In cases where continuous outcomes were reported as weighted mean differences or raw mean differences, these were recalculated into an SMD (Hedges’ g) using Comprehensive Meta-Analysis 3.0. Studies that reported the outcomes by the following: (a) psychedelics dose (Hasler et al. 2004; Wittmann et al. 2007) and (b) separately for sessions before and after cross-over (Griffiths et al. 2016; Griffiths et al. 2006; Ross et al. 2016), each outcome having their data combined and the mean of the SMD being used in analysis. When there was more than one outcome, the effect sizes were calculated for each one and then the averaged was used in the meta-analysis (Higgins and Green 2011).

We also extracted the number of participants (*N*), along with the number of trials/comparisons (*K*) from which the pooled effect size was derived. Additionally, all analyses were performed with a random-effect model, which considers both between-study and within-study variability. The heterogeneity was quantified using the *I*^2^ statistic, and categorized as low (*I*^2^ < 25%), moderate (*I*^2^ = 25–50%), or high (*I*^2^ > 50%). Other relevant study characteristics were also extracted, specifically with regard to dose and if psychotherapy support was offered.

Sub-group or secondary analyses of clinical response with respect to the type of psychedelic substance or population (patients with a mood disorder or healthy volunteers) were also undertaken. Safety and tolerability outcomes were not included in the meta-analysis due to marked heterogeneity in reporting. Instead, this information was extracted and narratively reviewed. Although there is a recognized difficulty in having appropriate blinding in studies with psychedelics, we assumed for the calculation of the JADAD score that an appropriate placebo could include substances that may induce some similar physiological and/or cognitive effect of psychedelics, such as methylphenidate, niacin, or low doses of the psychedelics. The potential impact of publication bias was assessed using fail-safe N and Egger’s regression test of the intercept. A statistically significant effect was regarded as a *p* value of < 0.05. The data was analyzed and figures prepared via Comprehensive Meta-Analysis 3.0.

## Results

### Systematic search results

**Fig. 1:**
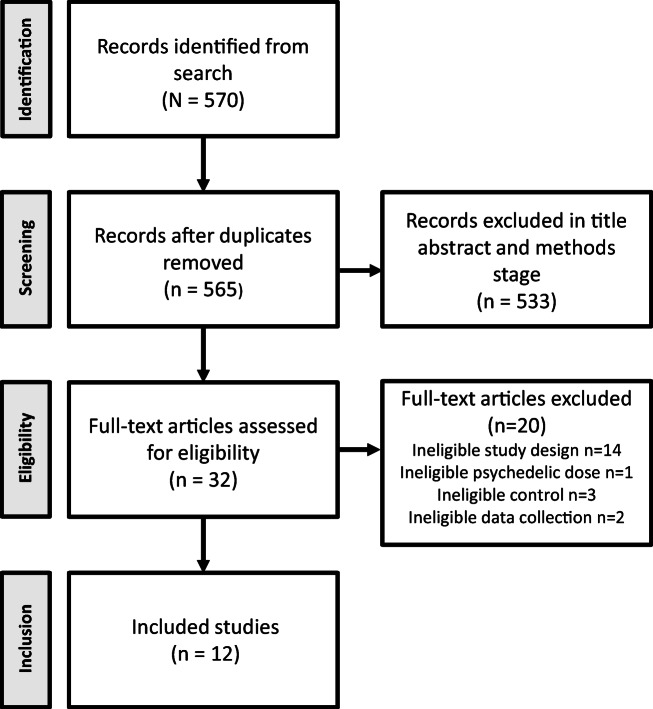
PRISMA flow diagram of systematic review and meta-analysis of classic serotonergic psychedelics for mood and depressive symptoms

The search returned 570 results, which was reduced to 565 after duplicates were removed. Title and abstract screening removed 533 articles, while 32 manuscripts were retrieved and reviewed in full. Of these, 14 were open-label clinical trials and consequently were not included in this meta-analysis (Bogenschutz et al. 2015; Carhart-Harris et al. 2018; Carhart-Harris et al. 2016a; Carhart-Harris et al. 2017; Carhart-Harris et al. 2011; Kaelen et al. 2018; Lyons and Carhart-Harris 2018a; Lyons and Carhart-Harris 2018b; Osorio Fde et al. 2015; Roseman et al. 2017; Sanches et al. 2016; Stroud et al. 2018). From the 18 double-blind trials selected, 6 were excluded because they did not meet the inclusion criteria. Specifically, 3 did not have a placebo-control design (Daumann et al. 2008; Gouzoulis-Mayfrank et al. 2005; Schmid and Liechti 2018); 1 clinical trial used micro-doses of LSD (Bershad et al. 2019); 1 trial measured outcomes only 2 h after treatment (Santos et al. 2007), and one clinical trial showed unpublished outcomes collected after 60 days of psychedelic dosing session (Griffiths et al. 2008). Thus, we were left 12 studies which met the criteria for inclusion. For the PRISMA flow diagram, see Fig. 1.

### Description of studies

For study attributes involving type of participants, mood diagnosis, number of treatment session, psychedelic doses, and placebo type, see Table 2. Of the 12 studies included, 8 used psilocybin (Griffiths et al. 2016; Griffiths et al. 2006; Grob et al. 2011; Hasler et al. 2004; Kometer et al. 2012; Kraehenmann et al. 2015; Ross et al. 2016; Wittmann et al. 2007), 3 used LSD (Dolder et al. 2016; Gasser et al. 2014; Schmid et al. 2015), and 1 used DMT in the form of ayahuasca (Palhano-Fontes et al. 2019). In summary, these involved 257 participants, made up of 124 healthy volunteers and 133 patients with mood disorders. All trials provided a single psychedelic administration by dose, with exception of Gasser et al. (2014), where LSD was administered twice.

**Table 2:** Study summary of clinical trials included in the meta-analysis

Study	Design	*N*	Subject	Psychedelic	Dose	Session^#^	Placebo
Gasser et al. 2014	CO	12	A and LT	LSD	200 μg	2	LSD 20 μg
Schmid et al. 2015	CO	16	Healthy	LSD	200 μg	1	Mannitol**
Dolder et al. 2016	CO	16	Healthy	LSD	100 μg	1	Mannitol**
Palhano-Fontes et al. 2019	P	29	TRD	Ayahuasca	360 μg/kg	1	Zinc sulfate
Hasler et al. 2004	CO	8	Healthy	Psilocybin	^&^115, 215 and 315 μg/k	4	Lactose
Wittmann et al. 2007	CO	12	Healthy	Psilocybin	115 and 250 μg/kg	2	Lactose
Griffiths et al. 2006	CO	30	Healthy	Psilocybin	429 μg/kg	1	Methylphenidate
Kometer et al. 2012	CO	17	Healthy	Psilocybin	215 μg/kg	1	Not specified
Kraehenmann et al. 2015	CO	25	Healthy	Psilocybin	160 μg/kg	1	Lactose
Griffiths et al. 2016	CO	51	A, D and LT	Psilocybin	314 and 429 μg/kg	1	P* 43 and 14 μg/kg
Ross et al. 2016	CO	29	A and LT	Psilocybin	300 μg/kg	1	Niacin
Grob et al. 2011	CO	12	A and LT	Psilocybin	200 μg/kg	1	Niacin

*CO*, cross-over; *P*, parallel; *N*, sample size; *A*, anxiety; *D*, depression; *TRD*, treatment-resistant depression; *LT*, life-threatening illness; *LSD*, lysergic acid diethylamide, P*, psilocybin

^#^Number of psychedelic sessions during the trial

^&^Low dose of 45 mg/kg of psilocybin was not included in the meta-analysis

^**^Information provided by the author, it does not have in the manuscript

All studies utilized a cross-over design, other than Palhano-Fontes et al. (2019), which used a parallel design. Ross et al. (2016), Gasser et al. (2014), Griffiths et al. (2006), and Griffiths et al. (2016) also included psychotherapy to support the psychedelic intervention. No clinical trials involving mescaline met the criteria for inclusion.

### Quality assessment of the included meta-analyses

For methodological quality scores of all the clinical trials included, see supplementary material Table S1. Two studies had a maximum score of 5/5: Ross et al. (2016) (psilocybin) and Palhano-Fontes et al. 2019 (ayahuasca). Of note, from 12 studies, only 2 trials described the randomization technique used while only 7 detailed participant withdrawal. The main issue related to studies quality was the blinding process. From 12 studies included, 3 did not present details regarding the placebo condition used. However, for two of them, we were able to obtain further information directly from the study authors (Table S1).

Few studies analyzed the integrity of the blinding process by questionnaires assessed in volunteers and/or via the research team (Gasser et al. 2014; Griffiths et al. 2006; Griffiths et al. 2016; Palhano-Fontes et al. 2019; Ross et al. 2016). Griffiths et al. (2006 and 2016), while Palhano-Fontes et al. (2019) related success in the blinding process, whereas Gasser et al. (2014) and Ross et al. (2016) did not appear to achieve success in blindness despite the use as placebo a low dose of psychedelic (LSD) and niacin, respectively.

Although the participant expectancy about the study is not part of the JADAD scale, it is relevant to analyze as it can play a significant role both in blinding and in participants and evaluators responses. From 12 studies, 3 detailed some strategies aiming to reduce these expectancies, such as the use of instructional sets, multiples evaluators, naïve volunteers, individual, and not groups experiments, parallel study designer (Griffiths et al. 2006; Griffiths et al. 2016; Palhano-Fontes et al. 2019). However, no studies used expectancy measures as a co-factor in statistical analysis of clinical response.

### Mood state and depressive symptom outcomes

#### Mood state

Meta-analyses were conducted on the measures of negative mood state of healthy volunteers and also in patients with a mood disorder, separately. Only one study was included in the systematic review, analyzing medium-term mood changes; therefore, it was not possible to undertake a meta-analysis for this time-point (Grob et al. 2011).

**Fig. 2:**
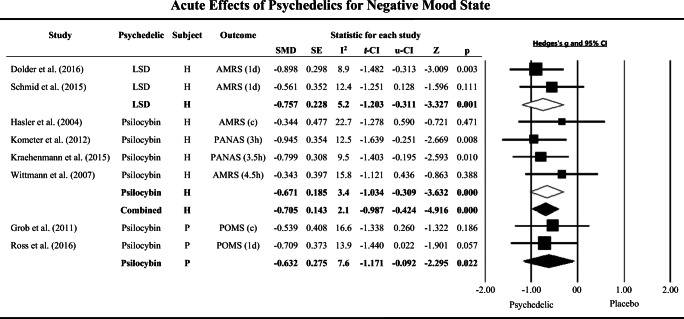
The effect size (SMD) of acute clinical effects of classic serotonergic psychedelic and placebo treatments on negative mood state in healthy volunteers and patients with mood disorders, shown as Hedges’ g with 95% confidence interval. Negative Hedges’ g indicates favor of psychedelics. Squares represent study effect sizes; open diamonds represent effect sizes of sub-group analyses by drug (lysergic acid diethylamide [LSD] or psilocybin); closed diamonds represent overall effect sizes for healthy volunteers (H) or mood disorder patients (P). The sizes of squares and diamonds are proportional to the SMD. Combined: polled LSD and psilocybin. d: day and h: hours. (c) The following instruments were grouped and the mean of SMD was used in analysis: Grob et al. (2011), Profile of Mood States (POMS) of 6 h and 1 day. Hasler et al. (2004), Adjective Mood Rating Scale (AMRS) of 4.5 h and 1 day. PANAS: Positive and Negative Affect. SE, standard error; l-IC, low confidence interval; u-CI, up confidence interval; *I*^2^, heterogeneity across studies (%); Z, *z* value; p, *p* value

Meta-analysis of acute measures of mood state, collected between 3 h and 1 day after treatment, showed a moderate clinical effect size of psychedelics in the reduction of negative mood when compared to placebo in both healthy participants (*N* = 103, *K* = 6, SMD = − 0.705, CIs − 0.987 to − 0.424, *p* < 0.01; *I*^2^ = 2.1%) and patients with a mood disorder (*N* = 41, *K* = 2, SMD = − 0.632, CIs − 1.171 to − 0.092, *p* = 0.022; *I*^2^ = 7.6%), with low variance across studies (Healthy volunteers: Dolder et al. 2016; Hasler et al. 2004; Kometer et al. 2012; Kraehenmann et al. 2015; Schmid et al. 2015; Wittmann et al. 2007, patients: Grob et al. 2011; Ross et al. 2016) (Fig. 2 and supplementary material table S2). No study was located providing data on acute mood state changes after ayahuasca treatment.

Sub-analysis by psychedelic drug in healthy volunteers revealed a highly significant effect with a moderate effect size for both LSD (*N* = 32, *K* = 2, SMD = − 0.757, CIs − 1.203 to − 0.311, *p* = 0.001; *I*^2^ = 5.2%) (Dolder et al. 2016; Schmid et al. 2015) and psilocybin in negative mood reduction with low variability across studies (*N* = 62, *K* = 4, SMD = − 0.671, CIs − 1.034 to − 0.309, *p* < 0.001; *I*^2^ = 3.4%) (Hasler et al. 2004; Kometer et al. 2012; Kraehenmann et al. 2015; Wittmann et al. 2007) (Fig. 2 and table S2).

Moreover, the meta-analysis of long-term measures of mood state, between 16 and 60 days after treatment, showed that psilocybin also had a moderate long-term effects in reduction of negative mood in patients with a mood disorder, with low heterogeneity across trials (*N* = 110, *K* = 3, SMD = − 0.495, CIs − 0.829 to − 0.161, *p* = 0.004; *I*^2^ = 2.9%) (Griffiths et al. 2006; Ross et al. 2016) (Table S2). No studies examined long-term changes on mood state in healthy participants, and after LSD or ayahuasca treatment.

### Depressive symptoms

Three meta-analyses were conducted with measures of depressive symptoms assessed by depression symptom rating scales from patients with a mood disorder: acute effects, medium-term, and longer-term clinical effects.

**Fig. 3:**
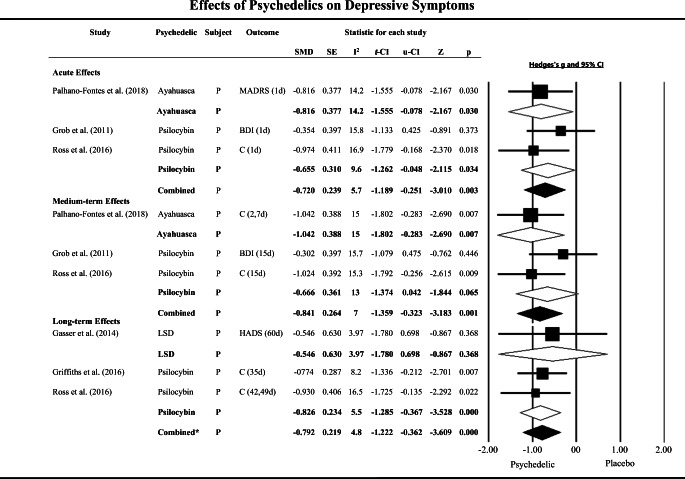
Effect size (SMD) of acute, medium-term, and long-term clinical effects of classic serotonergic psychedelics vs placebo treatments on depressive symptoms of patients with mood disorders (P), shown as Hedges’ g with 95% confidence interval. A negative Hedges’ g indicates favor of psychedelics. Squares represent study effect sizes; open diamonds represent effect sizes of sub-group analyses by drug (psilocybin, lysergic acid diethylamide [LSD], and ayahuasca); closed diamonds represent overall effect sizes of each time-point (acute, medium term, and long term). The sizes of squares and diamonds are proportional to the SMD. Combined: pooled ayahuasca and psilocybin. Combined*: pooled LSD and psilocybin. d: day and h: hours. C: The score of the following instruments were grouped and the mean of SMD was used in analysis, for Ross et al. (2016) and Griffiths et al. (2016), Beck Depression Inventory (BDI) and Hospital Anxiety and Depression Scale (HADS); Palhano-Fontes et al. (2019), Hamilton Depression Rating Scale and Montgomery–Åsberg Depression Rating Scale (MADRS). SE, standard error; l-IC, low confidence interval; u-CI, up confidence interval; *I*^2^, heterogeneity across studies (%); Z, *z* value; p, *p* value

The meta-analysis of acute effects (between 3 h and 1 day) on depressive symptoms showed a significant and moderate clinical effect size of psychedelics (psilocybin and ayahuasca) for reduction of depressive symptoms. Again, low heterogeneity was observed across studies (*N* = 70, *K* = 3, SMD = − 0.720, CIs − 1.189 to − 0.251, *p* = 0.003; *I*^2^ = 5.7%) (Grob et al. 2011; Palhano-Fontes et al. 2019; Ross et al. 2016) (Fig. 3 and table S2). A sub-analysis of the individual psychedelics revealed that psilocybin had a significant and moderate clinical effect size on the reduction of depressive symptoms in patients with a mood disorder, with low variance across studies (*N* = 41, *K* = 2, SMD = − 0.665, CIs − 1.262 to − 0.048, *p* = 0.034; *I*^2^ = 9.6%) (Grob et al. 2011; Ross et al. 2016) (Fig. 3 and table S2). There were no clinical trials located involving the acute assessment of LSD’s effects on depressive symptoms.

We found a significant and large effect of classic psychedelics (psilocybin and ayahuasca) in the medium-term assessment of depressive symptoms, with low heterogeneity across studies (*N* = 70, *K* = 3, SMD = − 0.841, CIs − 1.359 to − 0.323, *p* = 0.001; *I*^2^ = 7.0%) (Grob et al. 2011; Palhano-Fontes et al. 2019; Ross et al. 2016) (Fig. 3 and table S2). However, a secondary analysis of psilocybin studies showed a marginally non-significant effect for reduction of depression between 7 and 15 days after treatment (*N* = 41, *K* = 2, SMD = − 0.666, CIs − 1.374 to − 0.042, *p* = 0.065; *I*^2^ = 13%) (Grob et al. 2011; Ross et al. 2016) (Fig. 3 and table S2). We can note that the medium-term clinical effects of classic psychedelics on depressive symptoms were mainly driven by data from the ayahuasca study.

The assessment of the longer-term (16 to 60 days) effect of psychedelics on the reduction of depressive symptoms revealed a highly significant effect with a moderate to large effect size (*N* = 92, *K* = 3, SMD = − 0.792, CIs − 1.222 to − 0.362, *p* < 0.001; *I*^2^ = 4.8%) (Gasser et al. 2014; Griffiths et al. 2006; Ross et al. 2016) (Fig. 3 and table S2). The sub-analysis of psilocybin trials only also showed a large clinical effect in the reduction of depressive symptoms in patients with mood disorders, with low heterogeneity between studies (*N* = 80, *K* = 2, SMD = − 0.826, CIs − 1.285 to − 0.367, *p* < 0.001; *I*^2^ = 5.5%) (Griffiths et al. 2016; Ross et al. 2016) (Fig. 3 and table S2).

### Safety and tolerability

From the 12 studies included, 3 did not detail data pertaining to safety or tolerability of the psychedelic used (Kometer et al. 2012; Kraehenmann et al. 2015; Wittmann et al. 2007). In general, the other clinical trials reported that classic serotonergic psychedelics were well-tolerated. Acute psychological side-effects induced by psychedelics in the included studies were mainly mild anxiety episodes, tearing/crying, nausea, vomit, headache, and slight sympathomimetic effect, such as increase in blood pressure, heart rate, and pupil size, and rare episodes of paranoia was related. No study participants were noted as requiring pharmacological intervention to address these side-effects. Long-term studies did not indicate any persistent anxiety, suicidal crisis, or psychotic state.

### Publication bias

With concern of publication bias, the fail-safe *N* of this meta-analysis is 114 (*N* = 12, *Z* = − 6.339, *p* < 0.001). There would need to be 9.5 missing studies for every observed study for the effect to be nullified. Egger’s regression test of the intercept also did not report publication bias (B0 = − 0.191, IC = − 2.541 to 2.158, *t* = 0. 181, df = 10, 1-tailed *p* = 0.429). For the funnel plot of publication bias, see supplementary material figure S1.

## Discussion

This meta-analysis combined and evaluated data from 12 double-blind RCTs investigating the efficacy of classic serotonergic psychedelics on mood state and depressive symptoms, between 3 h and 60 days after administration, in patients with mood disorders, and healthy volunteers, separately. We observed a significant moderate effect size for reduction of acute negative mood outcomes in healthy volunteers, compared to placebo, as well as significant moderate effects sizes for acute and long-term reductions of negative mood state in patients with mood disorders. For depressive symptoms, a significant large effect size was detected from a medium-term assessment, and a moderate effect size for both acute and long-term outcomes was observed for patients, compared to placebo.

Trials that assessed mood state response to psychedelics were in general conducted in healthy volunteers and mainly assessed acute outcomes. In this context, secondary analysis by psychedelic drug revealed moderate effect sizes for both psilocybin and LSD, with a slightly larger effect size for LSD. A significant moderate effect size in studies assessing the acute reduction of negative mood also was observed to patients in psilocybin trials. An analysis of longer-term data (16–60 days after treatment) indicated that psilocybin maintains its response on negative mood reduction of patients, with a moderate effect size compared to placebo. In this meta-analysis, it was not possible to undertake a meta-analysis for medium-term mood changes due to insufficient clinical trials.

It is important study the effects of classic psychedelics in both healthy volunteers and those with diagnosed mood disorders because as their neurobiology is distinct, and the psychobiological responses to drugs can be different between these populations (Galvão et al. 2018). It is essential to understand if any mood-elevating effects are evident in healthy participants, and to be able to compare this with the response of those with clinical depression. For note, it is usual that clinical trials select only one of these groups, with few studies assessing parallels groups of patients and healthy controls (de Almeida et al. 2019; Galvão et al. 2018; Galvão-Coelho et al. 2020). This meta-analysis provides a data analysis of negative mood in both healthy participants and patients with mood disorders, to differentially assess both populations.

The larger effect size of LSD in acute reductions of negative mood, in comparison to psilocybin, may be due to differing pharmacokinetic profiles (Libânio Osorio Marta 2019). While the onset of psychedelic effects is typically faster with psilocybin, around 30 min vs 1 h for LSD, the total duration of psilocybin acute effects are substantially shorter, at around 3.5 h, compared with 8 to 12 h for LSD, depending on dose (Araujo et al. 2015; Dolder et al. 2017). However, pharmacokinetic data in this area remains limited. Besides classic serotonergic psychedelics, few double-blinded, placebo-controlled clinical trials have aimed to analyze acute mood changes in response to substances with psychedelic action (Krystal et al. 2006; Micallef et al. 2003). Furthermore, positive results for ketamine treatment in reduction of negative mood are for example observed 1 day after its administration, when compared with midazolam treatment (Grunebaum et al. 2018).

Psychedelics, specifically psilocybin and ayahuasca, demonstrated a moderate effect size in the acute reduction of depressive symptoms compared to placebo. The fast onset of therapeutic response is a key characteristic of new potential “fast-acting antidepressants,” which aside from classic psychedelics also may include ketamine (Corriger and Pickering 2019; Ly et al. 2018). Animal models reveal that the biological therapeutic actions of antidepressants may in part be mediated via increased neuroplasticity through the expression of brain-derived neurotropic factor (BDNF) and its tyrosine kinase receptors type B in the prefrontal cortex and hippocampus (Mannari et al. 2008; Pilar-Cuéllar et al. 2012). Therefore, the delay in the therapeutic onset may potentially correlate with the time required for the elevation of the BDNF (Jesulola et al. 2018). While fast-acting antidepressants also induce neuroplasticity by BDNF increases, both in vitro and in vivo, this occurs through a pathway different and faster than that of antidepressants, involving the mammalian target of rapamycin (Ly et al. 2018). Moreover, it has been suggested that substances with psychedelic effect are able to reframe negative memories, which is in contrast to standard antidepressant pharmacotherapy in which new information is processed with a positive bias (Harmer et al. 2017; Kometer et al. 2012).

The present meta-analysis also revealed a large effect size of psychedelics (psilocybin, LSD, and ayahuasca) compared to placebo, in the reduction of depressive symptoms in medium-term investigations, that is, between 2 and 15 days after treatment, which was evident from the ayahuasca study. A moderate effect size was seen for other psychedelics (LSD and psilocybin) in the longer-term analysis (between 16 and 60 days after interventions). Though it should be noted that this was due to the assessment time-points employed, and not necessarily to do with the length of ongoing antidepressant effect, another agent with a shorter alacrity of response than standard antidepressants, ketamine, suggests a similar temporal effects profile, with the strongest responses occurring between 1 and 2 weeks post-treatment (Corriger and Pickering 2019; Sanacora et al. 2017). Furthermore, a recent meta-analysis showed stronger effect sizes of classic psychedelics in reduction of depressive symptoms on days 7 and 21 after treatment comparing with baseline scores (Romeo et al. 2020).

Sub-analysis of trials using psilocybin with patients with a mood disorder and life-threatening disease identified moderate acute and large long-term effects of this substance in the reduction of depressive symptoms, when compared to placebo. These results are highly encouraging of further research with this group, as a recent meta-analysis of the use of antidepressants for cancer patients reported no indication of effects superior to placebo (Ostuzzi et al. 2018). The use of psychedelics in this population may extend beyond addressing affective symptoms, having a potentially specialized role in assisting with existential psychological distress involving mortality. However, it is important to highlight that mood disorders in these patients are often a comorbidity of the life-threatening disease, and the depressive symptoms can be expressed in different ways for instance from the depressive patients where the depression is the main pathology.

Although anxiety symptoms are often present in mood disorders, from 12 selected studies in systematic review, only 6 measured anxiety, and due the heterogeneity of these studies, it was not possible to conduct a meta-analysis with them (LSD, Gasser et al. 2014; Psylocibin; Grob et al. 2011; Kometer et al. 2012; Kraehenmann et al. 2015; Ross et al. 2016; Griffiths et al. 2016). However, it is important to highlight the importance of conducting this analysis when the data becomes available.

An additionally important point in this context is the use of “psychotherapy-assisted” applications of psychedelics. This approach may not only be safer (and more ethical); it may also provide a potentially stronger therapeutic effect. The psychedelic-assisted therapy, analyzed by studies that used MDMA and classic psychedelics, showed stronger clinical improvement of different mood and anxiety disorder than placebo (Luoma et al. 2020). This would however in the future be advised to be assessed via controlled research comparing psychedelic interventions alone and in combination with psychological assistance to determine if any additive or synergistic effect was evident. Moreover, such comparison would determine whether either intervention provides better management of any psychological distress which may emerge during consumption of the psychedelic.

It is also important to note that despite the low number of studies included in meta-analysis, low statistical heterogeneity was observed across clinical trials and no publication bias was detected. Moreover, in general, the included clinical trials indicated that classic serotonergic psychedelics are well-tolerated, although more reporting precision on adverse effects and longer safety follow-ups are recommended in future studies. Acute psychological and psychological side-effects were mainly mild anxiety episodes and sympathomimetic effects, such as increases in blood pressure, heart rate, and pupil size, which were short-lived and did not require pharmacological intervention.

There are several critical challenges recognized in conducting robust double-blind studies involving psychedelics. As revealed in our assessment of the methodological quality of the studies reviewed, a significant issue was evident regarding the presence of adequate blinding. In particular, some studies did not report any details regarding the placebo used, while others used a placebo which may or may not have active effects. To address the issue of sufficient blinding, some trials have used “active” placebos, such as low doses of psychedelics or methylphenidate and niacin, which can induce mild physiologic and cognitive changes. This is a well-needed methodological advancement; however, some studies still find issues in blinding process even after this approach. Therefore, we must consider that unblinding is, at least in part, responsible for the magnitude of the effect size provided in this meta-analysis. Moreover, the expectancy of participants and evaluators about the treatment can also modulate the results. Despite 3 studies detailing strategies to deal with this potential expectation, none included expectancy measures as a covariate in statistical analysis of clinical response, and this should be considered in future studies.

Another issue of concern is the conducting of trials with multiple sessions of psychedelics or cross-over designs. Most included studies in this review used a cross-over study design, introducing possible limitations related to potential carry-over effects, as this raises the chance of the first session experience increasing the expectation bias of next session (or resulting in a carry-over effect if the active intervention is firstly used). Moreover, how the data analysis is communicated in cross-over studies should be better considered by the researchers. For instance, separate analyses of the first and second cross-over treatment administered, rather than having these both results analyzed together, are potentially a clearer method. Additionally, some included clinical trials did not provide adequate detail about the randomization strategy used and how participant withdrawals were handled in the analysis, making risk of bias assessment for these domains difficult. Further studies in this area are recommended to ensure adequate reporting of randomization procedures in line with international reporting guidelines (Higgins and Green 2011). Other limitations should also be taken in account in this meta-analysis: the small sample sizes of the included studies, the high heterogeneity in study design and population, multiple psychedelic doses, variety of outcome scales used, and different time-points assessed. For the last point, we acknowledge that analyzing the long-term effects may not entirely be methodologically sound due to the long duration between substance use and data collection. Therefore, future studies should consider these aspects with the aim of improving the quality of trials (Johnson et al. 2008).

In summation, methodological weaknesses aside, our meta-analysis provides encouraging evidence for the potential use of classic serotonergic psychedelics in the reduction of both negative mood state and depressive symptoms. While there are currently limited studies that have investigated some of these agents (in particular ayahuasca), the promising results of this review support the need for ongoing and more robust research in this emerging field to further explore the effect of psychedelics in adults with depression.

## Supplementary information

ESM 1 (RTF 1513 kb)

ESM 2 (DOCX 18.9 kb)

ESM 3 (DOCX 20.5 kb)
