# Supplementary material for: Classic serotonergic psychedelics for mood and depressive symptoms: a meta-analysis of mood disorder patients and healthy participants
Source: Psychopharmacology (Berl). 2021 Jan 11;238(2):341–54. doi: 10.1007/s00213-020-05719-1 (PMC7826317; doi:10.1007/s00213-020-05719-1)
Supplement: Supplementary file 1 — (RTF 1513 kb) [file 213_2020_5719_MOESM1_ESM.rtf]

Figure S1. Funnel plot of publication bias of double-blind placebo-controlled clinical trials that tested the effects of classic serotoninergic psychedelics on mood and depressive symptoms in health volunteers and mood disorder patients. Circles denote studies
