# Supplementary material for: Classic serotonergic psychedelics for mood and depressive symptoms: a meta-analysis of mood disorder patients and healthy participants
Source: Psychopharmacology (Berl). 2021 Jan 11;238(2):341–54. doi: 10.1007/s00213-020-05719-1 (PMC7826317; doi:10.1007/s00213-020-05719-1)
Supplement: Supplementary file 2 — (DOCX 18.9 kb) [file 213_2020_5719_MOESM2_ESM.docx]

**Supplementary material**

**Table S1** Quality of eligible clinical trials assessed using the Jadad scale

|  | | | | | |  |  |
| --- | --- | --- | --- | --- | --- | --- | --- |
| **JADAD* Score** | | | | | | | |
| **Author** | **Randomized (+1)** | **Appropriate Randomized (+1/-1)** | **Double-Blind (DB) (+1)** | **Appropriate Double-Blind**  **DB (+1/-1)** | **Withdrawals (+1)** | | **Score (5)** |
| Gasser et al. 2014 | 1 | 0 | 1 | 1 | 1 | | 4 |
| Schmid et al. 2015 | 1 | 0 | 1 | 0 | 1 | | 3 |
| Dolder et al. 2016 | 1 | 0 | 1 | 0 | 1 | | 3 |
| Palhano-Fontes et al. 2019 | 1 | 1 | 1 | 1 | 1 | | 5 |
| Hasler et al. 2004 | 1 | 0 | 1 | 0 | 0 | | 2 |
| Wittmann et al. 2007 | 1 | 0 | 1 | 0 | 0 | | 2 |
| Griffiths et al. 2006 | 1 | 0 | 1 | 1 | 1 | | 4 |
| Kometer et al. 2012 | 1 | 0 | 1 | 0 | 0 | | 2 |
| Kraehenmann et al. 2015 | 1 | 0 | 1 | 0 | 0 | | 2 |
| Griffiths et al. 2016 | 1 | 0 | 1 | 1 | 1 | | 4 |
| Ross et al. 2016 | 1 | 1 | 1 | 1 | 1 | | 5 |
| Grob et al. 2011 | 1 | 0 | 1 | 1 | 0 | | 3 |

*Jadad, A. R., Moore, R. A., Carroll, D., Jenkinson, C., Reynolds, D. J. M., Gavaghan, D. J., & McQuay, H. J. (1996). *Assessing the quality of reports of randomized clinical trials: Is blinding necessary? Controlled Clinical Trials, 17(1), 1–12.* doi:10.1016/0197-2456(95)00134-4
