# Supplementary material for: Classic serotonergic psychedelics for mood and depressive symptoms: a meta-analysis of mood disorder patients and healthy participants
Source: Psychopharmacology (Berl). 2021 Jan 11;238(2):341–54. doi: 10.1007/s00213-020-05719-1 (PMC7826317; doi:10.1007/s00213-020-05719-1)
Supplement: Supplementary file 3 — (DOCX 20.5 kb) [file 213_2020_5719_MOESM3_ESM.docx]

**Supplementary material**

**Table S2** The effect size of classic serotonergic psychedelics^.

| **Effect**  **Time** | **Volunteer** | **Psychedelics**  **Type** | **Hedges´s g** | **K** | **SE** | **I^2^** | **CI Lower** | **CI Upper** | **z-Value** | **p-Value** |
| --- | --- | --- | --- | --- | --- | --- | --- | --- | --- | --- |
| *Negative Mood* | |  |  |  |  |  |  |  |  |  |
| Acute | Healthy | Combined* | -0.705 | 6 | 0.143 | 2.1 | -0.987 | -0.424 | -4.916 | 0.000 |
|  |  | LSD | -0.757 | 2 | 0.228 | 5.2 | -1.203 | -0.311 | -3.327 | 0.001 |
|  |  | Psilocybin | -0.671 | 4 | 0.185 | 3.4 | -1.034 | -0.309 | -3.632 | 0.000 |
|  | Patients | Psilocybin | -0.632 | 2 | 0.275 | 7.6 | -1.171 | -0.092 | -2.295 | 0.022 |
| Long-term | Patients | Psilocybin | -0.495 | 3 | 0.171 | 2.9 | -0.829 | -0.161 | -2.903 | 0.004 |
|  |  |  |  |  |  |  |  |  |  |  |
| *Depressive symptoms* | |  |  |  |  |  |  |  |  |  |
| Acute | Patients | Combined** | -0.720 | 3 | 0.239 | 5.7 | -1.189 | -0.251 | -3.010 | 0.003 |
|  |  | Psilocybin | -0.655 | 2 | 0.310 | 9.6 | -1.262 | -0.048 | -2.115 | 0.034 |
| Medium-term | Patients |  |  |  |  |  |  |  |  |  |
|  |  | Combined** | -0.841 | 3 | 0.264 | 7 | -1.359 | -0.323 | -3.183 | 0.001 |
|  |  | Psilocybin | -0.666 | 2 | 0.361 | 13 | -1.374 | 0.042 | -1.844 | 0.065 |
| Long-term | Patients |  |  |  |  |  |  |  |  |  |
|  |  | Combined*** | -0.792 | 3 | 0.219 | 4.8 | -1.222 | -0.362 | -3.609 | 0.000 |
|  |  | Psilocybin | -0.826 | 2 | 0.234 | 5.5 | -1.285 | -0.367 | -3.528 | 0.000 |

^ On the reduction of negative mood state and depressive symptoms, compared to placebo, in healthy volunteers and patients with mood disorder. P-value ≤0.05 indicates significant difference between psychedelic and placebo treatment; Negative Hedges´s g (SMD) indicates favor of psychedelic; K: number of clinical trials; SE: Standard error; I^2^: heterogeneity across studies (%); CI: 95% confidence interval; Acute effects (3hs to 1 day after treatments); medium-term effects (2 to 15 days after treatments); Long-term clinical effects (16 to 60 days after treatments). Combined means that the following psychedelics were grouped and the mean of SMD was used in analysis, *: Lysergic acid diethylamide (LSD) and Psilocybin, **: Psilocybin and Ayahuasca, ***: Psilocybin and LSD.
